# Development and validation of ultrasound-based radiomics model to predict germline *BRCA* mutations in patients with breast cancer

**DOI:** 10.1186/s40644-024-00676-w

**Published:** 2024-02-29

**Authors:** Tingting Deng, Jianwen Liang, Cuiju Yan, Mengqian Ni, Huiling Xiang, Chunyan Li, Jinjing Ou, Qingguang Lin, Lixian Liu, Guoxue Tang, Rongzhen Luo, Xin An, Yi Gao, Xi Lin

**Affiliations:** 1 Department of Ultrasound, State Key Laboratory of Oncology in South China, Collaborative Innovation Center for Cancer Medicine, Sun Yat-Sen University Cancer Center, Guangzhou, 510060 China; 2 School of Biomedical Engineering, Shenzhen University Medical School, Shenzhen University, Shenzhen, 518000 China; 3 Department of Medical Oncology, State Key Laboratory of Oncology in South China, Collaborative Innovation Center for Cancer Medicine, Sun Yat-Sen University Cancer Center, Guangzhou, 510060 China; 4 Department of Ultrasound, Guangdong Second Provincial General Hospital, Guangzhou, 510060 China; 5 Guangdong Provincial Key Laboratory of Malignant Tumor Epigenetics and Gene Regulation, Department of Ultrasound, Sun Yat-Sen Memorial Hospital, Sun Yat-Sen University, Guangzhou, 510060 China; 6 Department of Pathology, State Key Laboratory of Oncology in South China, Collaborative Innovation Center for Cancer Medicine, Sun Yat-Sen University Cancer Center, Guangzhou, 510060 China

**Keywords:** Breast cancer, *BRCA*, Ultrasound, Radiomics, Nomogram

## Abstract

**Background:**

Identifying breast cancer (BC) patients with germline breast cancer susceptibility gene (g*BRCA*) mutation is important. The current criteria for germline testing for BC remain controversial. This study aimed to develop a nomogram incorporating ultrasound radiomic features and clinicopathological factors to predict g*BRCA* mutations in patients with BC.

**Materials and methods:**

In this retrospective study, 497 women with BC who underwent g*BRCA* genetic testing from March 2013 to May 2022 were included, including 348 for training (84 with and 264 without a g*BRCA* mutation) and 149 for validation(36 patients with and 113 without a g*BRCA* mutation). Factors associated with g*BRCA* mutations were identified to establish a clinicopathological model. Radiomics features were extracted from the intratumoral and peritumoral regions (3 mm and 5 mm) of each image. The least absolute shrinkage and selection operator regression algorithm was used to select the features and logistic regression analysis was used to construct three imaging models. Finally, a nomogram that combined clinicopathological and radiomics features was developed. The models were evaluated based on the area under the receiver operating characteristic curve (AUC), calibration, and clinical usefulness.

**Results:**

Age at diagnosis, family history of BC, personal history of other *BRCA*-related cancers, and human epidermal growth factor receptor 2 status were independent predictors of the clinicopathological model. The AUC of the imaging radiomics model combining intratumoral and peritumoral 3 mm areas in the validation set was 0.783 (95% confidence interval [CI]: 0.702—0.862), which showed the best performance among three imaging models. The nomogram yielded better performance than the clinicopathological model in validation sets (AUC: 0.824 [0.755—0.894] versus 0.659 [0.563—0.755], *p* = 0.007).

**Conclusion:**

The nomogram based on ultrasound images and clinicopathological factors performs well in predicting g*BRCA* mutations in BC patients and may help to improve clinical decisions about genetic testing.

**Supplementary Information:**

The online version contains supplementary material available at 10.1186/s40644-024-00676-w.

## Introduction

Breast cancer (BC) is the most common cancer and one of the leading causes of death from cancer among women globally [1]. The most prevalent and significant susceptibility gene of BC is the breast cancer susceptibility gene (*BRCA*), which includes *BRCA1* and *BRCA2* [2]. Knowledge of one’s germline *BRCA* (g*BRCA*) status has value for both the patient and her family. A therapeutic benefit exists for BC patients, because contralateral BC or ovarian cancer can be prevented by risk-reducing mastectomy and salpingo-oophorectomy [3]. Furthermore, with the advent of poly (ADP-ribose) polymerase inhibitor treatment, enabled treatment selection with improved outcomes [4]. For her family, it is possible to strengthen the gene screening of her close relatives.

Due to the increasing influence of gene mutations on BC surveillance, prevention, and treatment decisions, genetic testing is rapidly expanding in clinical practice [5]. However, the criteria for germline testing for BC remain controversial. The National Comprehensive Cancer Network guildline recommends genetic testing only for high-risk patients, which may exclude half of the cases that do not fit this criterion [6, 7]. In addition, genetic testing is time-consuming and expensive, routine genetic testing for the majority of or all BC patients may result in a large financial burden, ethical dilemmas, and other obstacles [8–10]. Therefore, before performing genetic testing, an accurate estimation of the probability of BC patients carrying a g*BRCA* mutation is crucial.

Recent studies [11–13] have demonstrated the viability and potential utility of radiomics as a technique for predicting the g*BRCA* status of cancer patients by utilizing demographic and clinicopathological features, pathology images, or magnetic resonance imaging (MRI) images. Biomedical images can contain information that reflects the underlying pathophysiology [14]. Over the last few years, radiomics has been used by oncologists and radiologists for diagnosis, therapy response assessment, and survival prediction in BC patients [15–17]. In addition, some studies [15, 18–20] have shown that combined intratumoral and peritumoral radiomics models have superior performance compared to intratumoral radiomics models alone. The peritumoral region refers to the adjacent parenchyma immediately surrounding the tumor. It may be considered to represent the tumor microenvironment and has biological importance in defining tumor behavior [21–23].

However, due to insufficient accuracy of clinical criteria or the limited number of cases included in some studies [11–13], and the higher costs and lower availability of MRI than other imaging modalities [24, 25], the existing risk prediction models for genetic testing do not fulfill the requirements of clinical practice. Therefore, there is an urgent need for a valid, accurate, and cost-effective model to predict g*BRCA* mutations. Ultrasound (US) is widely used to characterize breast lesions, because of its low cost, wide availability, real-time image analysis capabilities, and lack of ionizing radiation emission [26]. In addition, due to the high proportion of young Asian women with hereditary BC, given their relatively dense breasts, information obtained from US images may provide a reference for the subsequent genetic testing of this population. Several reports comparing morphological characteristics from US images between sporadic and *BRCA1*/*2*-related BC have been published [27–29]. However, to date, no studies have been published on the use of US images to predict the g*BRCA* mutation status of BC patients.

Hence, the purpose of this study was to develop a nomogram based on intratumoral and peritumoral US features, combined with clinicopathological factors, to predict the g*BRCA* mutation status of patients with BC.

## Methods

### Study population

The study protocol was approved by the Academic Ethics Committee of Sun Yat-sen University Cancer Center. Because of the retrospective nature of this study, the need for informed consent was waived.

The study participants were women diagnosed with BC who were recruited from the Sun Yat-sen University Cancer Center from March 2013 to May 2022. The following were the criteria for inclusion: (I) clear g*BRCA* gene test results, (II) patients who underwent breast ultrasound, and (III) pathologically confirmed BC. The exclusion criteria were (I) clinicopathological information was incomplete, (II) preoperative therapy (chemotherapy, radiotherapy, or incomplete resection), and (III) poor picture quality. There were 497 patients finally enrolled after the application of these criteria. The patients were divided into two sets at a ratio of 7:3. The training set included 348 women (84 with and 264 without a g*BRCA* mutation), while the validation set included 149 women (36 patients with and 113 without a g*BRCA* mutation).

### US image acquisition

All lesions underwent examination by breast US before the operation. US examinations were performed using high-frequency (7–18 MHz) linear array probes and a real-time US system. US was performed in two orthogonal planes, and the lesions’ characteristics were recorded. The US systems used included Logiq 9, Logiq E9, and Logiq S8 (GE Medical Systems, Waukesha, WI, USA); IU22 and EPIQ 7 (Philips Healthcare, Amsterdam, Netherlands); ACUSON Juniper, Sequoia, and S2000 (Siemens Healthineers, Erlangen, Germany); Aixplorer (Supersonic Imagine, Aix-en-Provence, France); Aplio 400 (Toshiba Medical Systems Corp, Tochigi, Japan); Aloka ProSound ALPHA 10 (Hitachi-Aloka Medical, Wallingford, CT, USA); Resona 7 T and DC-8 (Mindray Medical International, Shenzhen, China); and MyLab 70 (Esaote, Genoa, Italy) systems.

### g*BRCA *mutation status

Genomic DNA was extracted from patients’ peripheral blood. *BRCA1*/*2* gene fragments were sequenced by next-generation sequencing. Searches for mutations were limited to known deleterious mutations. To prevent possible dataset contamination [30], variants of uncertain signifcance (VUS) were excluded from the analysis. g*BRCA* genetic testing results were used as the gold standard.

### Image pre-processing, region of interest segmentation, and feature extraction

**Fig. 1:**
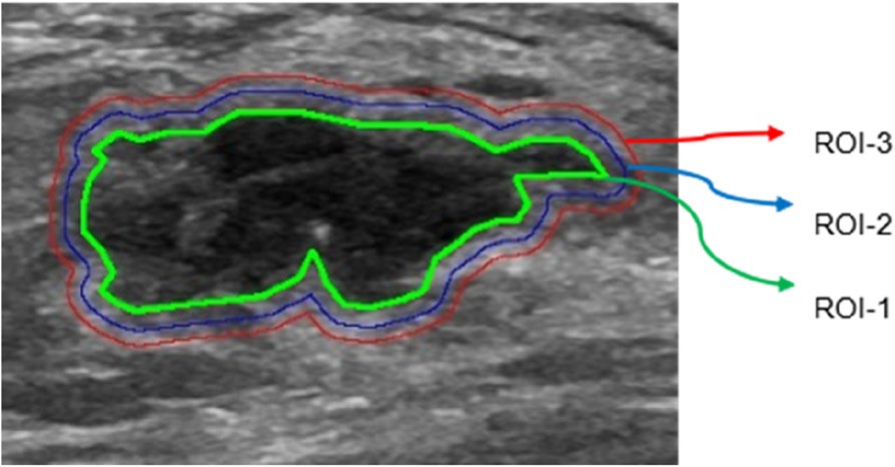
The regions of interest. ROI = region of interest; ROI-1 = intratumoral area; ROI-2 = 3 mm peritumoral region; ROI-3 = 5 mm peritumoral region

The BC lesions located in US B-mode images were manually delineated along the tumor edge by a radiologist (with 3 years of experience in breast imaging) as the region of interest (ROI-1). Another radiologist (with 10 years of experience in breast imaging) examined all of the ROIs. If the readings were discordant, agreement was arrived at by a joint review of the images. Neither physician was aware of the patient’s g*BRCA* mutation status. When there were multiple lesions in the image, the largest lesion was selected as the target lesion. Based on other previous imaging studies of the peri-tumor area of breast cancer [15, 19, 20], we decided to externally expand the peri-tumor area to 3 mm and 5 mm. The Opencv package of the Python program was used to semi-automatically segment the peritumoral area (ROI-2 and ROI-3, including the peritumoral parenchyma representing 3 mm and 5 mm extensions outward, respectively) (Fig. 1).

**Fig. 2:**
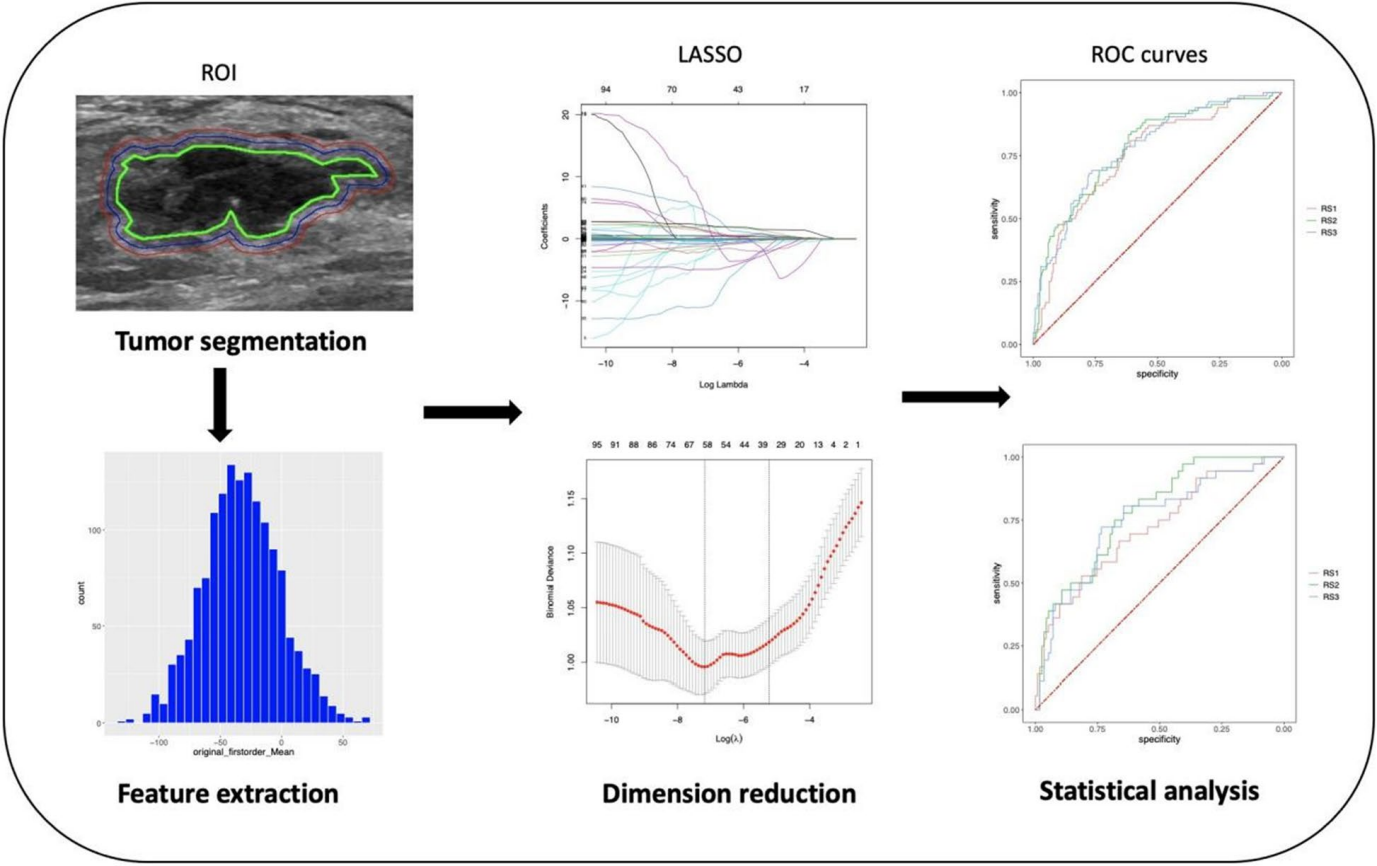
Overview of the radiomics modeling process. ROI = region of interest, LASSO = least absolute shrinkage and selection operator, ROC = receiver operating characteristics

This study used Z-score normalization to standardize features, aligning them to a mean of zero and a standard deviation of one, in order to remove the inherent bias introduced by multiple ultrasound systems before feature selection. There were 1,359 radiomic features, including first-order statistics, shape, gray-level co-occurrence matrix (GLCM), gray-level size zone matrix (GLSZM), gray-level dependence matrix (GLDM), Gy-level run length matrix (GLRLM), and neighborhood gray-tone difference matrix (NGTDM), that were extracted from three segmented regions (ROI-1, ROI-2, and ROI-3). These features were used for further analysis and regression modeling. More information about the standard radiomics workflow and model construction is shown in Fig. 2.

### Radiomics score

Imaging data were featured from the three ROIs using the t-test followed by the least absolute shrinkage and selection operator (LASSO) algorithm (Supplementary eFig. 1). The t-test was cited first to find the most discriminated features and the most useful predictive combination of data was used to create three radiomics signatures (RS1 for ROI-1, RS2 for ROI-1 and ROI-2, and RS3 for ROI-1 and ROI-3) by linear combination. Selection bias of LASSO may introduced due to the limited samples. To choose more robust features, the most suitable coefficient λ was selected through tenfold cross-validation. The final radiomics signature was obtained by logistic regression. Based on the combined radiomics signature, a radiomics score was calculated and presented in the training and validation sets. We calculated the area under the curve (AUC) for three models in the training and validation sets, and selected the model with the highest AUC as the final radiomics model.

### Development of the clinicopathological model

Baseline clinicopathological data were obtained from the medical records. Univariate logistic regression analysis was used to screen candidate variables in the training set, and variables with *p* < 0.200 were entered into a multivariate logistic regression analysis. Variables with *p* < 0.050 in the multivariate analysis were then included as independent predictors in the final clinicopathology model. After selecting clinicopathological features, a logistic regression model based on these features was constructed as the clinicopathological model.

### Development of the clinicopathology–radiomics signature and nomogram

To integrate radiomics and clinicopathological features, we constructed a combined clinicopathology-radiomics model. The t-test was also cited to select radiomics features first, and then we selected all radiomics features and all clinicopathological features identified by LASSO in the training set, and the penalty coefficient, λ, was also determined using ten-fold cross-validation. To prevent overfitting, we used a ridge regression model with a penalty coefficient of 0.05 and intuitively represented the model as a nomogram. In the nomogram, the threshold probability of a g*BRCA* mutation was determined based on the cutoff index. The likelihood that a patient would be projected to have a g*BRCA* mutation increased when it exceeded the threshold probability.

### Statistical analysis

Normalization was performed on radiomics features using z-score transformation. To assess the equivalence of patient demographic data between cohorts, normally distributed data were analyzed using an independent Student’s t-test, and non-normally distributed data expressed as medians were analyzed using the Mann–Whitney U test. Categorical variables were analyzed using a chi-square test. The predictive performance of the different models was evaluated using receiver operating characteristic (ROC) curves. The area under the ROC curve (AUC) and balanced sensitivity and specificity at the cutoff value were calculated. DeLong’s test was used to compare the AUCs between the models. Calibration curves and the Hosmer–Lemeshow (H–L) test were used to assess the calibration performance of the nomogram [31]. Decision curve analysis (DCA) was implemented to determine the clinical utility of the nomogram by quantifying the net benefits at different threshold probabilities [32]. Statistical analyses were performed using R software (version 4.0.4; R Foundation for Statistical Computing, Vienna, Austria). To address the issue of multicollinearity, the variance inflation factor (VIF) was calculated for each feature, and features with a VIF of > 10 were excluded. Statistical significance was defined as a two-sided *p*-value of < 0.050.

## Results

### Patient characteristics

Table 1 shows the clinicopathological characteristics of the 497 patients. The patients were divided into the g*BRCA* mutation group (*n* = 120, including 59 patients with a *BRCA1* mutation and 61 patients with a *BRCA2* mutation) and the non-g*BRCA* mutation group (*n* = 377). The prevalence of bilateral BC, a personal history of BC, a personal history of other *BRCA*-related cancers (ovarian cancer and pancreatic cancer), a family history of BC, and a family history of other *BRCA*-related cancers (ovarian cancer, pancreatic cancer, and prostate cancer); estrogen receptor (ER) status; and Ki67 and human epidermal growth factor receptor 2 (HER-2) status were significantly different between the g*BRCA* mutation group and the non-g*BRCA* mutation group (all *p* < 0.050). There were no statistically significant differences in age at diagnosis, menopausal status, the prevalence of multiple lesions, histological subtype, BC grade, lymph node status, or ER status between the *BRCA* mutation and non-*BRCA* mutation groups (all *p* > 0.050). No significant differences in patient characteristics were observed between the training and validation sets (Supplementary Table 1).

**Table 1:** Patient characteristics

Characteristics	g*BRCA* Mutation (*n* = 120)	Non-g*BRCA* Mutation (*n* = 377)	*p*
Age at diagnosis, year	41.96 ± 8.58	43.60 ± 10.1	0.107
Tumor size, mm	26.98 ± 11.0	25.86 ± 11.3	0.342
Menopausal status			0.743
Postmenopausal	95 (79.2)	291 (77.4)	
Premenopausal	25 (20.8)	86 (22.8)	
Multiple lesions			0.225
Yes	10 (8.3)	49 (13.0)	
No	110 (91.7)	328 (87.0)	
Bilateral breast cancer			0.038*
Yes	20 (16.7)	35 (9.3)	
No	100 (83.3)	342 (90.7)	
Personal history of breast cancer			0.009*
Yes	12 (10.0)	13 (3.4)	
No	108 (90.0)	364 (96.6)	
Personal history of other *BRCA*-related cancers			0.001*
Yes	9 (7.5)	5 (1.3)	
No	111 (92.5)	372 (98.7)	
Family history of breast cancer			< 0.001*
Yes	47 (39.2)	73 (19.4)	
No	73 (60.8)	304 (80.6)	
Family history of other *BRCA*-related cancers			0.021*
Yes	12 (10.0)	15 (4.0)	
No	108 (90.0)	362 (96.0)	
Histological subtype			0.110
Invasive	119 (99.2)	360 (95.5)	
Non-invasive	1(0.8)	17 (4.5)	
Grade			0.756
1 or 2	55 (45.8)	181 (48.0)	
3	65 (54.2)	196 (52.0)	
Lymph node status			0.862
Positive	60 (50.0)	194 (51.5)	
Negative	60 (50.0)	183 (48.5)	
ER status			0.035*
Positive	73 (60.8)	270 (71.6)	
Negative	47 (39.2)	107 (28.4)	
PR status			0.611
Positive	72 (60.0)	238 (63.1)	
Negative	48 (40.0)	139 (36.9)	
Ki67			0.011*
≥ 14%	115 (95.8)	328 (87.0)	
< 14%	5 (4.2)	49 (13.0)	
HER-2 status			0.005*
Positive	14 (11.7)	91 (24.1)	
Negative	106 (88.3)	286 (75.9)	

*BRCA* Breast cancer susceptibility gene, *ER* Estrogen receptor, *PR* Progesterone receptor, *HER-2* human epidermal growth factor receptor 2

Data are the mean ± standard deviation for continuous variables and patient numbers for categorical variables

^*^Significance at *p* < 0.050

### Development and validation of radiomics signatures

In total, 1,359 radiomics features were extracted from three ROIs and were selected by the LASSO algorithm. Moreover, radiomics signatures (RS1, RS2, and RS3) were constructed by logistic regression. The radiomics score calculation formula is presented in Supplementary Table 2.

The AUC for RS1 was 0.754 (95% confidence interval [CI], 0.695—0.812) in the training set, and 0.718 (95% CI, 0.619—0.817) in the validation set. The AUC for RS2 was 0.783 (95% CI, 0.727—0.839) in the training set and 0.782 (95% CI, 0.702—0.862) in the validation set. The AUC for RS3 was 0.779 (95% CI, 0.723—0.835) in the training set and 0.745 (95% CI, 0.650—0.840) in the validation set (Table 2).

**Table 2:** Prediction performance of three imaging radiomics models in the training and validation sets

Model	Accuracy (%)	Sensitivity (%)	Specificity (%)	AUC (95% CI)
Training set				
Intratumor	66.4	77.4	62.9	0.754 (0.695, 0.812)
Combined 3 mm	67.2	77.4	64.0	0.783 (0.727, 0.839)
Combined 5 mm	66.7	77.4	63.3	0.779 (0.723, 0.835)
Validation set				
Intratumor	57.0	72.2	52.2	0.718 (0.619, 0.817)
Combined 3 mm	65.1	80.6	60.2	0.782 (0.702, 0.862)
Combined 5 mm	67.1	75.0	64.6	0.745 (0.650, 0.840)

*AUC* Area under the receiver operating characteristic curve, *CI* Confidence interval, combined 3 mm, 3 mm intratumor and peritumor region; combined 5 mm, 5 mm intratumor and peritumor region

There were no statistically significant differences between different RSs (*p* > 0.05) according to DeLong’s test (Supplementary eFig. 2). As RS2 showed the best performance in the training and validation sets, with an AUC of 0.783 (95% CI, 0.727—0.839) in the training set and an AUC of 0.782 (95% CI, 0.702—0.862) in the validation set, it was selected as the final radiomics model for subsequent analyses.

### Development and validation of a clinicopathological model and nomogram

**Fig. 3:**
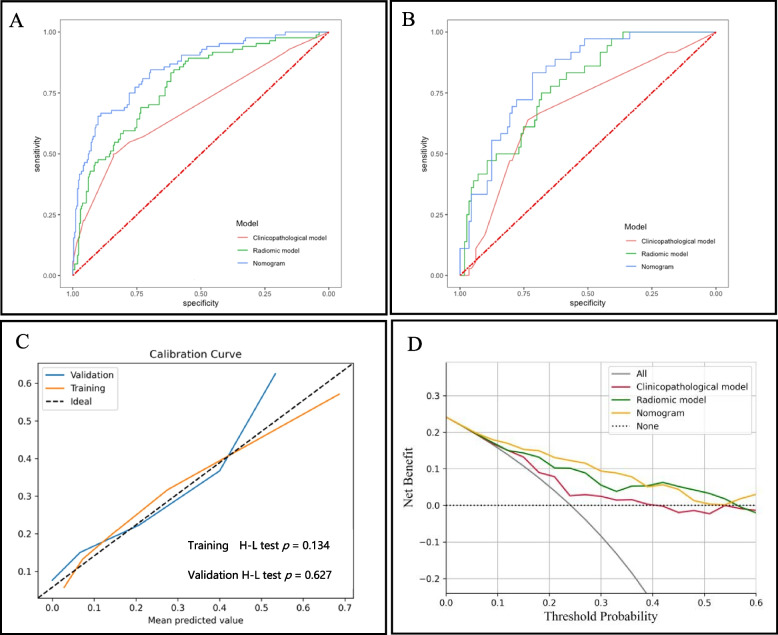
Results of the multivariate logistic regression model. **A** ROC curves of the training set. **B** ROC curves of the validation set. **C** Calibration curve of the combined model. **D** DCA figure of the three models of the validation set. ROC = receiver operating characteristics, H–L test = Hosmer–Lemeshow test; DCA = decision curve analysis

The clinicopathological characteristics were analyzed by univariate and multivariate logistics (Table 3). After multivariate logistic regression analysis, age at diagnosis, a personal history of other *BRCA*-related cancers, a family history of BC, and HER-2 status remained significant factors for *BRCA* mutations (*p* < 0.050). The AUCs of the training and validation sets were 0.708 (95% CI, 0.642—0.774) and 0.659 (95% CI, 0.563—0.755), respectively (Table 4; Fig. 3A, B). The AUC of radiomics model is higher than the clinicopathological model (0.782 vs 0.659), of borderline statistical significance (*p* = 0.056).

**Table 3:** Univariate and multivariable logistic regression models for the prediction of g*BRCA* mutations in patients with breast cancer

**Variables**	**Univariate**			**Multivariate**	
	**OR (95% CI)**	***p***		**OR (95% CI)**	***p***
Age at diagnosis	0.98 (0.95, 1.00)	0.093		0.97 (0.94, 0.99)	0.021*
Tumor size	1.00 (0.98, 1.03)	0.687			
Menopausal status	0.95 (0.52, 1.69)	0.870			
Multiple lesions	0.62 (0.24, 1.37)	0.264			
Bilateral breast cancer	1.68 (0.80, 3.38)	0.158		1.13 (0.36, 3.32)	0.826
Personal history of breast cancer	2.31 (0.81, 6.22)	0.101		3.38 (0.76, 15.23)	0.108
Personal history of other *BRCA*-related cancers	16.65 (2.63, 321.40)	0.011*		48.10 (5.51, 1143.00)	0.002*
Family history of breast cancer	3.37 (1.98, 5.73)	< 0.001*		4.44 (2.47, 8.09)	< 0.001*
Family history of other *BRCA*-related cancers	1.33 (0.41, 3.70)	0.603			
Histological subtype	4.65 (0.91, 84.87)	0.141		4.46 (0.72, 88.37)	0.182
Grade	1.04 (0.64, 1.70)	0.883			
Lymph node status	0.93 (0.57, 1.52)	0.762			
ER status	1.71 (1.02, 2.85)	0.039*		1.59 (0.90, 2.82)	0.112
PR status	1.00 (0.60, 1.64)	0.986			
Ki67	3.06 (1.18, 10.46)	0.040*		3.60 (1.14, 16.20)	0.051
HER-2 status	2.00 (1.05, 4.08)	0.043*		2.15 (1.08, 4.61)	0.038*

*BRCA* Breast cancer susceptibility gene, *ER* Estrogen receptor, *PR* Progesterone receptor, *HER-2* Human epidermal growth factor receptor 2

^*^Significance at *p* < 0.050

**Table 4:** Predictive performance of the three models in the training and validation sets

Model	Accuracy (%)	Sensitivity (%)	Specificity (%)	AUC (95% CI)
Training set				
Clinicopathological model	62.4	69.0	60.2	0.708 (0.642, 0.774)
Radiomics score	67.2	77.4	64.0	0.783 (0.727, 0.839)
Nomogram	74.7	81.0	72.7	0.850 (0.803, 0.898)
Validation set				
Clinicopathological model	53.7	69.4	48.7	0.659 (0.563, 0.755)
Radiomics score	65.1	80.6	60.2	0.782 (0.702, 0.862)
Nomogram	72.5	83.3	69.0	0.824 (0.755, 0.894)

*AUC* Area under the receiver operating characteristic curve, *CI* Confidence interval

**Fig. 4:**
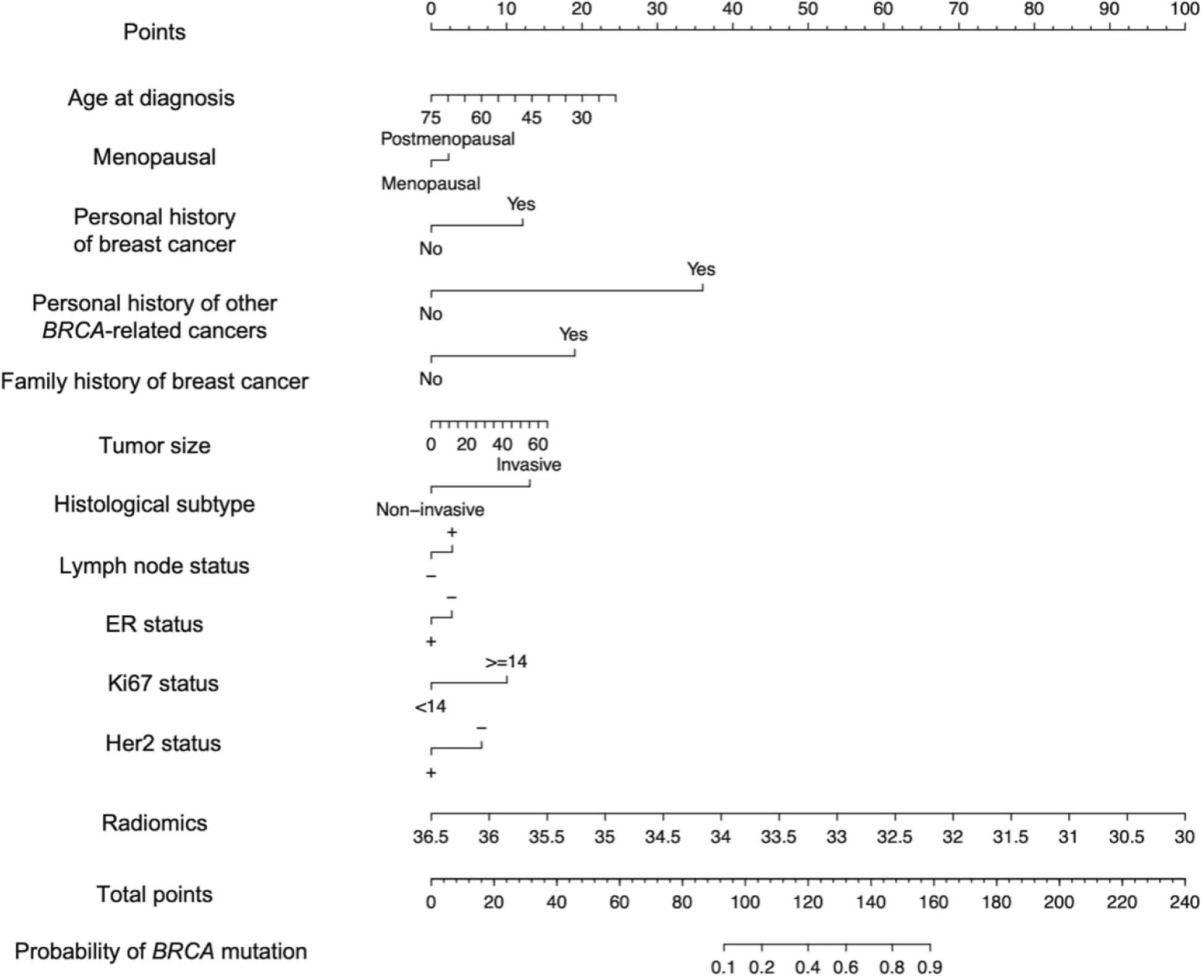
Nomogram constructed based on the combined model. Each point that corresponds to each variable is on the uppermost point scale. The sum of all points is referred to as the total points. The point total projected on the bottom scale indicates the probability of a g*BRCA* mutation in breast cancer patients. *BRCA* = breast cancer susceptibility gene, ER = estrogen receptor, PR = progesterone receptor, HER2 = human epidermal growth factor receptor 2

A nomogram was developed based on all of the radiomics features and clinicopathologic predictors (Fig. 4). In the nomogram, age at diagnosis, menopausal status, tumor size, personal history of BC, personal history of other *BRCA*-related cancers, family history of BC, histological type, lymph node status, ER status, Ki67 status, and HER-2 status were all independent predictors of *BRCA* mutations in BC patients (Fig. 4). The thresholding of the nomogram output probabilities at a value of 0.250. The selection of the optimal threshold is detailed in Supplementary Table 3. As shown in  Table 4 and  Fig. 3, the AUCs of the nomogram were significantly larger than those of the clinicopathological model in both the training set (0.850 vs. 0.708, *p* < 0.001) and the validation set (0.824 vs. 0.659, *p* = 0.007). In addition, the nomogram had better predictive accuracy than the radiomics score (training set: 0.850 vs. 0.783, *p* = 0.009; validation set: 0.824 vs. 0.782, *p* = 0.316). As shown in Fig. 3C, the majority of the calibration curves followed a diagonal line for both the training set (H–L test *p* = 0.134) and the validation set (H–L test *p* = 0.627), indicating reliable risk estimates of the nomogram. The DCA curves also revealed an improvement of the nomogram than clinicopathological model in the validation set (Fig. 3D).

## Discussion

As germline *BRCA1/2* testing has an established role in risk management, it is increasingly important in therapy selection [4]. For more precise individualized treatment, it is necessary to identify whether a BC patient has a g*BRCA* mutation. Therefore, in this retrospective study, we developed and internally validated a US-based nomogram integrating clinicopathological variables. The nomogram showed an AUC of 0.824 in the validation set for predicting the g*BRCA* mutation status of patients with BC. It may be used as pre-screening tool to improve the cost-effectiveness of genetic testing before it is performed, thus contributing to precision medicine.

Although the methodology for detecting genetic variants has greatly improved, genetic testing is usually time-consuming, has a high cost, and may be limited by the availability of suitable samples. US has the advantage of low cost, widespread availability, real-time image analysis capabilities, and lack of ionizing radiation, particularly adapt to women with dense breasts [26]. Thus, a method to predict gene mutations quickly and inexpensively from US images may be beneficial for the treatment of patients with BC, given the importance and impact of these mutations.

Radiomics is a method that extracts large amounts of data through high-throughput medical imaging. It is able to transform images into measurable features for further objective and quantitative analysis of the biological characteristics of diseases [14]. Previous studies have shown that image-feature-based radiomics has great value for diagnosis, therapy response assessment, and survival prediction in BC patients [15–17]. Furthermore, some studies have revealed that combined intratumoral and peritumoral radiomics models have superior efficacy compared to intratumoral radiomics models alone [15, 18, 33]. Our findings revealed that a combined intratumoral and peritumoral 3 mm region radiomics signature was the most optimal model for predicting the g*BRCA* mutation status of BC patients, as it showed the highest AUCs of 0.783 and 0.782 in the training and validation sets, respectively, and which is consistent with previous results [18, 33]. Our results suggested that radiomics signatures from the peritumoral area provide a reference for the accurate prediction of g*BRCA* mutation in breast lesions. But the biological mechanism underlying the peritumoral imaging features and their association with gene mutation remains unclear. Further studies are warranted to determine how the underlying biological changes were reflected by peritumor imaging features.

In multivariate regression analyses, we found that the risk factors significantly associated with g*BRCA* mutation status identified in this study were consistent with previously published findings from Asian countries [34–38]. These factors included younger age at diagnosis, ER-negative status, HER-2 negative status, and the presence of a family member with BC or ovarian cancer. Compared to women from Western nations, Asian women are diagnosed with BC much earlier in life [39, 40]. Furthermore, previous studies have shown that BC grade is also a significant risk factor for g*BRCA* mutations in high-risk BC patients [41, 42], but it was not significant in our study. This discrepancy may have been caused by the sample, as there was a small number of patients with mutations in our study.

In our study, the nomogram developed for g*BRCA* mutation prediction demonstrated favorable prediction and yielded AUCs of 0.850 and 0.824 in the training and validation sets, respectively. The nomogram had better predictive performance than the clinicopathological model for g*BRCA* mutations (*p* < 0.050). The findings of the present study indicated that radiomics can be used to assist g*BRCA* mutation prediction based on ultrasound in BC. Radiomics models based on MRI and histopathology images have previously been developed to predict g*BRCA* mutations in patients with BC [11, 43], but the sample sizes of these studies were small, only 16 or 22 patients with *BRCA* mutation. To our knowledge, the nomogram in this study is the first available ultrasound radiomics model based on intratumoral and peritumoral features for g*BRCA* prediction in BC patients.

However, there are some limitations of this study. First, this study was a single-center retrospective study, and the sample size was relatively small. Second, precise modeling depends upon the implementation of accurate and rapid segmentation of tumor. However, manual segmentation employed in this study is experience-dependent, time- and energy-consuming. In addition, to ensure data integrity and cleanness, patients with VUSs were excluded from model construction in our study. This could affect the applicability of the model to real-world scenarios, we will focus on this group of patients and optimise the existing models in the future study. Furthermore, the examination of gene mutations in BC patients in this study was restricted to the *BRCA* mutations. *BRCA1* or *BRCA2* mutations that were not examined, and mutations in additional relevant susceptibility genes (e.g., *PALB2*) were not included in this study. Future prospective, multimodal US imaging, multicenter studies with larger populations are needed to further improve the performance of the model.

## Conclusion

In conclusion, we have developed and compared the performance of clinicopathological, radiomics, and nomogram models for predicting g*BRCA* mutations in patients with BC. The nomogram based on US images and clinicopathological information outperformed the clinicopathological and radiomics models in predicting g*BRCA* mutations in patients with BC, providing valuable information for g*BRCA* mutation in BC and clinical decisions about genetic testing.

### Supplementary Information

**Supplementary Material 1.**

## Data Availability

The datasets generated or analyzed during the study are not publicly available due to institutional regulations but are available from the corresponding author on reasonable request.

## Abbreviations

AUC: Area under the curve
BC: Breast cancer
*BRCA*: Breast cancer susceptibility gene
CI: Confidence interval
DCA: Decision curve analysis
ER: Estrogen receptor
*gBRCA*: Germline breast cancer susceptibility gene
GLCM: Gray level co-occurrence matrix
GLDM: Gray level dependence matrix
GLRLM: Gray level run length matrix
GLSZM: Gray level size zone matrix
HER-2: Human epidermal growth factor receptor 2
H–L test: Hosmer–Lemeshow test
LASSO: Least absolute shrinkage and selection operator
MRI: Magnetic resonance imaging
NGTDM: Neighborhood gray-tone difference matrix
OR: Odds radio
PARP: poly ADP-ribose polymerase
PR: Progesterone receptor
RF: Radiomics feature
ROC: Receiver operating characteristic
RS: Radiomics signature
ROI: Region of interest
US: Ultrasound
VIF: Variance inflation factor
WT: Wavelet transform
*PALB2*: Partner and localizer of *BRCA2*
