# Supplementary material for: Development and validation of ultrasound-based radiomics model to predict germline BRCA mutations in patients with breast cancer
Source: Cancer Imaging. 2024 Feb 29;24:31. doi: 10.1186/s40644-024-00676-w (PMC10905812; doi:10.1186/s40644-024-00676-w)
Supplement: Supplementary file 1 — Supplementary Material 1. [file 40644_2024_676_MOESM1_ESM.docx]

**SUPPLEMENTARY MATERIALS**

**eTable 1. Patient Characteristics**

| **Characteristics** | **Training set**  **(n = 348)** | **Validation set**  **(n = 149)** | ***p*** |
| --- | --- | --- | --- |
| Age at diagnosis, year | 43.31 ± 9.81 | 42.97 ± 9.62 | 0.726 |
| Tumor size, mm | 26.41 ± 11.00 | 25.49 ± 11.52 | 0.403 |
| Menopausal status |  |  | 0.261 |
| Postmenopausal | 83 (23.9) | 28 (18.8) |  |
| Premenopausal | 265 (76.1) | 121 (91.2) |  |
| Multiple lesions |  |  | 0.719 |
| Yes | 43 (12.4) | 16 (10.7) |  |
| No | 305 (87.6) | 133 (89.3) |  |
| Bilateral breast cancer |  |  | 0.119 |
| Yes | 44 (12.6) | 11 (7.4) |  |
| No | 304 (87.4) | 138 (92.6) |  |
| Personal history of breast cancer |  |  | 0.656 |
| Yes | 19 (5.5) | 6 (4.0) |  |
| No | 329 (94.5) | 143 (96.0) |  |
| Personal history of other *BRCA* related cancers |  |  | 0.68 |
| Yes | 11 (3.2) | 3 (2.0) |  |
| No | 337 (96.8) | 146 (98.0) |  |
| Family history of breast cancer |  |  | 0.136 |
| Yes | 77 (22.1) | 43 (28.9) |  |
| No | 271 (77.9) | 106 (71.1) |  |
| Family history of other *BRCA* related cancers |  |  | 0.262 |
| Yes | 22 (6.3) | 5 (3.4) |  |
| No | 326 (93.7) | 144 (96.6) |  |
| Histological subtype |  |  | 0.639 |
| Invasive | 334 (96.0) | 145 (97.3) |  |
| Non-invasive | 14 (4.0) | 4 (2.7) |  |
| Grade |  |  | 0.352 |
| 1+2 | 160 (46.0) | 76 (51.0) |  |
| 3 | 188 (54.0) | 73 (49.0) |  |
| Lymph nodes status |  |  | 0.102 |
| Positive | 169 (48.6) | 85 (57.0) |  |
| Negative | 179 (51.4) | 64 (43.0) |  |
| ER status |  |  | 0.778 |
| Positive | 242 (69.5) | 101 (67.8) |  |
| Negative | 106 (30.5) | 48 (32.2) |  |
| PR status |  |  | 0.622 |
| Positive | 220 (63.2) | 90 (60.4) |  |
| Negative | 128 (36.8) | 59 (39.6) |  |
| Ki67 |  |  | 0.828 |
| ≥14% | 309 (88.8) | 134 (89.9) |  |
| <14% | 39 (11.2) | 15 (10.1) |  |
| HER-2 status |  |  | 0.232 |
| Positive | 79 (22.7) | 26 (17.4) |  |
| Negative | 269 (77.3) | 123 (82.6) |  |

Data are the mean ± standard deviation for continuous variables and patient numbers for categorical variables. *Significance at *p* < 0.050. *BRCA* = breast cancer susceptibility gene, ER = estrogen receptor, PR = progesterone receptor, HER-2 = human epidermal growth factor receptor 2

**eTable 2. Essential Radiomics Features and Formula Composition**

| **Model** | **Intercept/feature name** | **Regression coefficient** |
| --- | --- | --- |
| **RS1** | **Intercept=7.7791** | **β** |
| RF1 | original_firstorder_InterquartileRange | -1.271E-02 |
| RF2 | original_firstorder_Kurtosis | 1.638E-02 |
| RF3 | original_firstorder_RootMeanSquared | -2.195E-03 |
| RF4 | original_glcm_Autocorrelation | 1.158E-03 |
| RF5 | original_glcm_JointAverage | -3.589E-02 |
| RF6 | original_glcm_Correlation | -1.518E+00 |
| RF7 | original_glcm_SumSquares | 8.259E-03 |
| RF8 | original_glszm_ZoneEntropy | -7.986E-01 |
| RF9 | original_gldm_GrayLevelVariance | -1.227E-03 |
| RF10 | original_gldm_LargeDependenceHighGrayLevelEmphasis | -1.054E-04 |
| RF11 | original_ngtdm_Contrast | -5.361E+00 |
| RF12 | wavelet.LH_firstorder_Mean | 3.384E-01 |
| RF13 | wavelet.LH_glcm_Imc2 | -2.362E+00 |
| RF14 | wavelet.LH_glrlm_RunEntropy | 8.333E-01 |
| RF15 | wavelet.LH_gldm_DependenceEntropy | -1.795E-01 |
| RF16 | wavelet.LH_ngtdm_Busyness | 2.860E-01 |
| RF17 | wavelet.LH_ngtdm_Complexity | -1.398E-05 |
| RF18 | wavelet.HL_firstorder_Mean | 1.498E+00 |
| RF19 | wavelet.LL_firstorder_90Percentile | -1.781E-03 |
| RF20 | wavelet.LL_firstorder_InterquartileRange | -6.603E-03 |
| RF21 | wavelet.LL_firstorder_MeanAbsoluteDeviation | -1.109E-02 |
| RF22 | wavelet.LL_firstorder_Skewness | -3.919E-01 |
| RF23 | wavelet.LL_firstorder_Variance | -1.023E-05 |
| RF24 | wavelet.LL_glcm_SumSquares | 2.202E-03 |
| RF25 | wavelet.LL_glszm_LargeAreaLowGrayLevelEmphasis | -1.094E-02 |
| RF26 | wavelet.LL_gldm_GrayLevelVariance | -3.314E-04 |
| RF27 | wavelet.LL_gldm_LargeDependenceHighGrayLevelEmphasis | -4.791E-05 |
| RF28 | wavelet.LL_gldm_LargeDependenceLowGrayLevelEmphasis | -3.014E-01 |
| RF29 | wavelet.LL_ngtdm_Contrast | -3.539E+00 |
| **RS2** | **Intercept=41.2012** | **β** |
| RF1 | original_glcm_Imc2 | 4.120E+01 |
| RF2 | wavelet.LH_firstorder_Mean | -9.122E+00 |
| RF3 | wavelet.LH_glcm_Imc2 | 4.131E-01 |
| RF4 | wavelet.LH_ngtdm_Busyness | -2.625E+00 |
| RF5 | wavelet.HL_firstorder_Mean | 3.122E-01 |
| RF6 | wavelet.LL_firstorder_InterquartileRange | 7.173E-01 |
| RF7 | wavelet.LL_firstorder_RobustMeanAbsoluteDeviation | -4.440E-03 |
| RF8 | wavelet.LL_glszm_LargeAreaLowGrayLevelEmphasis | -4.425E-03 |
| RF9 | wavelet.LL_gldm_LargeDependenceLowGrayLevelEmphasis | -8.281E-03 |
| RF10 | diagnostics_Image.original_Mean.3mm | -4.023E-01 |
| RF11 | original_firstorder_Median.3mm | -1.638E-02 |
| RF12 | wavelet.LH_firstorder_RootMeanSquared.3mm | -1.394E-02 |
| RF13 | wavelet.HL_firstorder_Median.3mm | -1.318E-01 |
| RF14 | wavelet.HL_glcm_Imc2.3mm | -5.856E-01 |
| RF15 | wavelet.HL_glszm_ZoneEntropy.3mm | 1.859E+00 |
| RF16 | wavelet.HH_glcm_ClusterProminence.3mm | 1.751E+00 |
| **RS3** | **Intercept=-3.3931** | **β** |
| RF1 | original_glcm_Imc2 | -4.639E+00 |
| RF2 | wavelet.LH_firstorder_Mean | 4.987E-01 |
| RF3 | wavelet.LH_glcm_Imc2 | -3.880E+00 |
| RF4 | wavelet.LH_ngtdm_Busyness | 4.177E-01 |
| RF5 | wavelet.HL_firstorder_Mean | 1.166E+00 |
| RF6 | wavelet.LL_firstorder_RobustMeanAbsoluteDeviation | -2.307E-02 |
| RF7 | wavelet.LL_glszm_LargeAreaLowGrayLevelEmphasis | -1.047E-02 |
| RF8 | diagnostics_Image.original_Mean.5mm | -1.461E-02 |
| RF9 | original_firstorder_Median.5mm | -1.072E-02 |
| RF10 | original_gldm_DependenceEntropy.5mm | -6.090E-01 |
| RF11 | wavelet.HL_firstorder_Median.5mm | -7.737E-01 |
| RF12 | wavelet.HL_glcm_Correlation.5mm | -1.052E+00 |
| RF13 | wavelet.HL_glcm_Imc2.5mm | 7.292E-02 |
| RF14 | wavelet.HL_glszm_ZoneEntropy.5mm | 1.631E+00 |
| RF15 | wavelet.HH_glcm_ClusterProminence.5mm | -4.524E-06 |
| RF16 | wavelet.HH_glszm_ZoneEntropy.5mm | 1.523E+00 |

Abbreviations, β, Regression coefficient; RF = radiomics feature, RS1 = radiomic signature selected from the intratumoral area; RS2 = combined radiomic signature selected from the intratumoral and peritumoral 3mm area; RS3 = combined radiomic signature selected from the intratumoral and peritumoral 5mm area.

**eTable 3. Performance of Nomogram at Different Thresholds**

| **Thresholds** | **Sensitivity (%)** | **Specificity (%)** | **Accuracy (%)** |
| --- | --- | --- | --- |
| 0.050 | 100.0 | 9.8 | 31.6 |
| 0.100 | 97.6 | 26.1 | 43.4 |
| 0.150 | 94.0 | 45.5 | 57.2 |
| 0.200 | 85.7 | 62.5 | 68.1 |
| 0.250 | 81.0 | 72.7 | 74.7 |
| 0.300 | 67.9 | 81.1 | 77.9 |
| 0.350 | 66.7 | 87.9 | 82.8 |
| 0.400 | 59.5 | 91.3 | 83.6 |
| 0.450 | 47.6 | 93.9 | 82.8 |
| 0.500 | 42.9 | 96.6 | 83.6 |


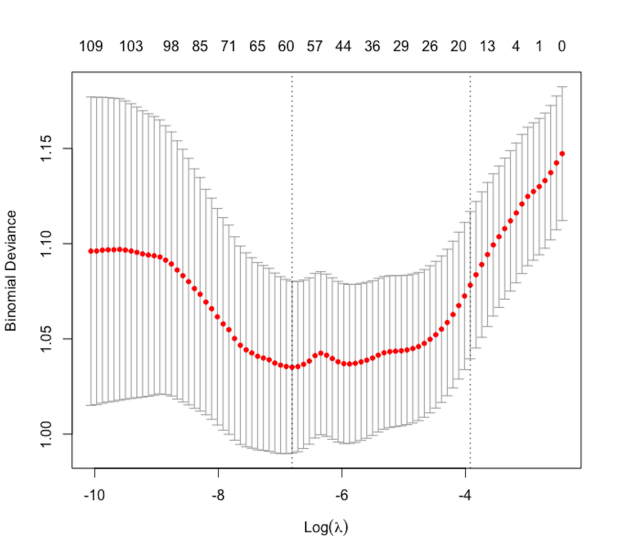

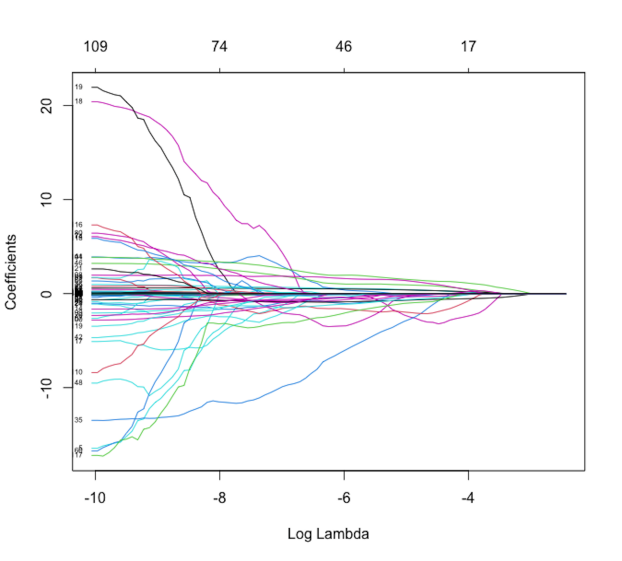

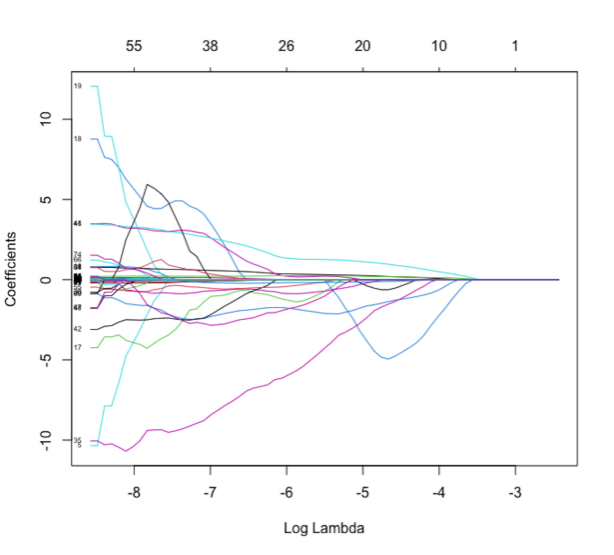

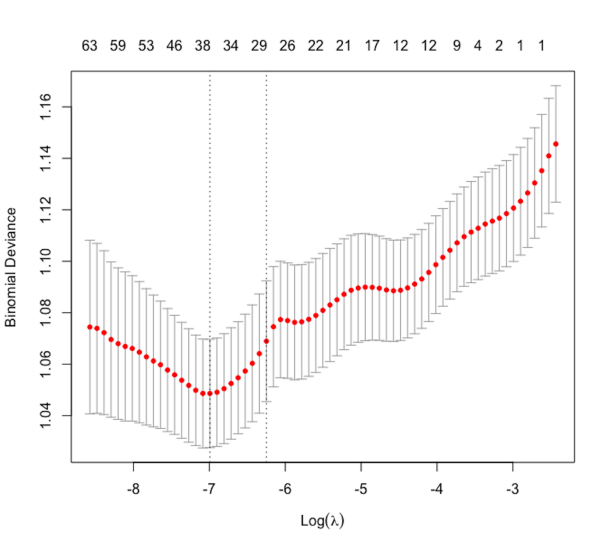

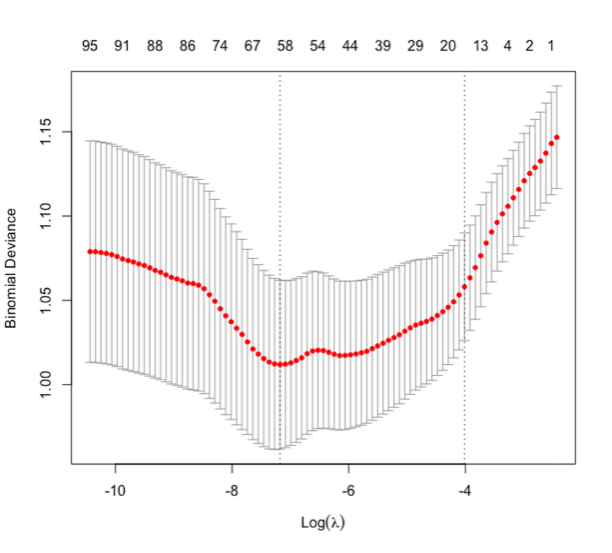

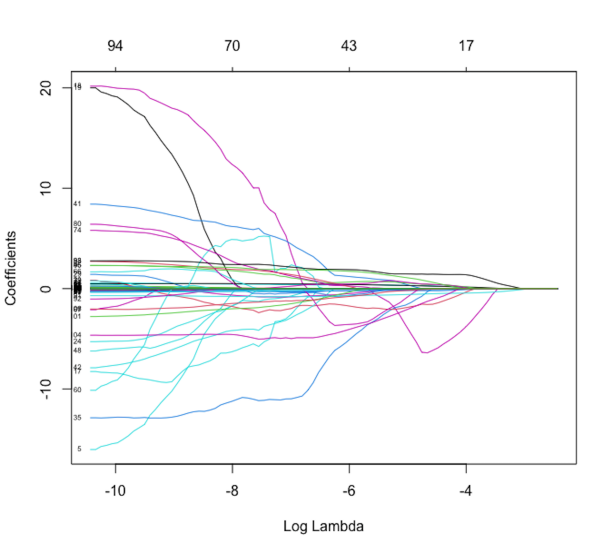


C

D

B

A

E

F

**eFigure 1. Identification of essential radiomic features of the intratumoral and peritumoral area with LASSO regression analysis.** Select radiomic features from the intratumoral area (A–B), intratumoral and peritumoral 3mm area (C–D), and intratumoral and peritumoral 5mm area (E–F) with LASSO. LASSO, Least absolute shrinkage and selection operator.


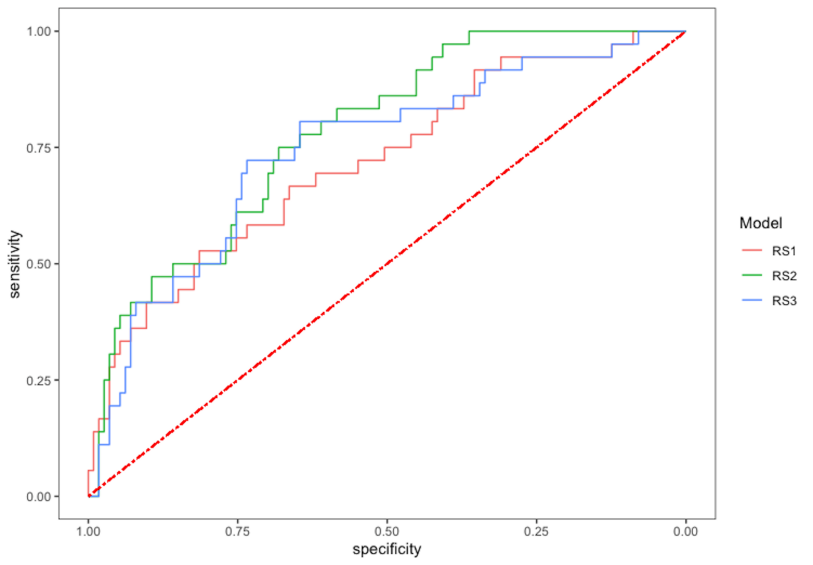

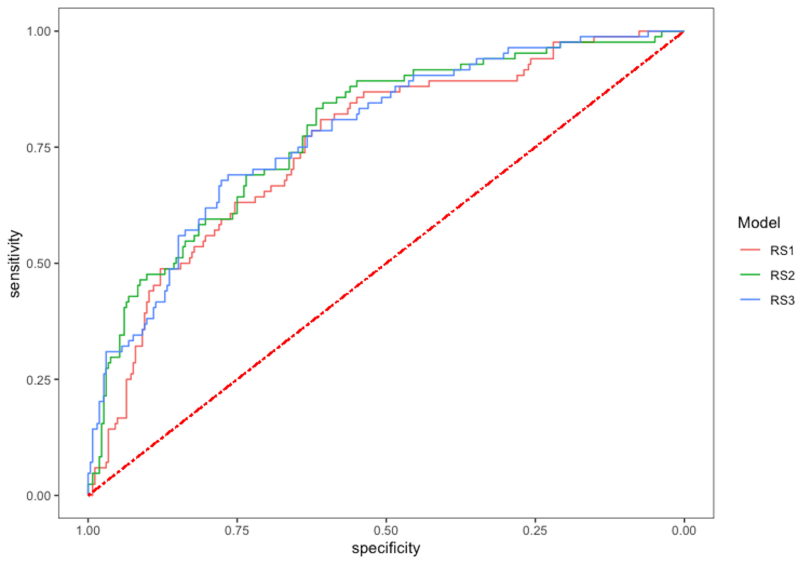


B

A

**eFigure 2. ROC curves of the three radiomic models in the development and validation.** Three imaging radiomics models for predicting BRCA germline mutation with receiver operating characteristic curve analysis in the training set **(A)**, validation set **(B)**.
